# Additively Manufactured Pneumatically Driven Skin Electrodes

**DOI:** 10.3390/ma11010019

**Published:** 2017-12-23

**Authors:** Martin Schubert, Martin Schmidt, Paul Wolter, Hagen Malberg, Sebastian Zaunseder, Karlheinz Bock

**Affiliations:** 1 Electronics Packaging Laboratory, Technische Universität Dresden, 01069 Dresden, Germany; wolterpa@avt.et.tu-dresden.de (P.W.); karlheinz.bock@tu-dresden.de (K.B.); 2 Institute of Biomedical Engineering, Technische Universität Dresden, 01307 Dresden, Germany; martin_schmidt@tu-dresden.de (M.S.); hagen.malberg@tu-dresden.de (H.M.); sebastian.zaunseder@tu-dresden.de (S.Z.)

**Keywords:** additive processing, 3D printing, on demand skin electrode, expandable electrode, electro dermal activity

## Abstract

Telemedicine focuses on improving the quality of health care, particularly in out-of-hospital settings. One of the most important applications is the continuous remote monitoring of vital parameters. Long-term monitoring of biopotentials requires skin-electrodes. State-of-the-art electrodes such as Ag/AgCl wet electrodes lead, especially during long-term application, to complications, e.g., skin irritations. This paper presents a low-cost, on-demand electrode approach for future long-term applications. The fully printed module comprises a polymeric substrate with electrodes on a flexible membrane, which establishes skin contact only for short time in case of measurement. The membranes that produce airtight seals for pressure chambers can be pneumatically dilated and pressed onto the skin to ensure good contact, and subsequently retracted. The dilatation depends on the pressure and membrane thickness, which has been tested up to 150 kPa. The electrodes were fabricated in screen and inkjet printing technology, and compared during exemplary electrodermal activity measurement (EDA). The results show less amplitude compared to conventional EDA electrodes but similar behavior. Because of the manufacturing process the module enables high individuality for future applications.

## 1. Introduction

One focus of telemedicine is to provide health care in out-of-hospital settings. To that end, long-term remote patient monitoring, i.e., recording of vital signs like respiratory rate, electrocardiogram (ECG) or electrodermal activity (EDA), and interactive services for patient care have become available in recent years. Such developments can improve the quality of a patient’s treatment by providing immediate access to healthcare. In particular, areas with high patient-to-doctor distance can benefit from such concepts [1]. However, even medically well-supplied areas can benefit, because long-term monitoring adds valuable information, whe compared to intermittent physical examinations by medical doctors. Besides, health care expenses can be reduced by gaining vital parameters remotely, without consulting a doctor [2].

The recording of biopotentials, i.e., electrical signals, is one essential aspect of remote monitoring systems [3,4]. To monitor biopotentials in telemedical settings, skin electrodes are used. Typically, skin electrodes can be roughly classified into wet and dry electrodes [5]. The conventional Ag/AgCl wet electrodes are widely used in the clinical environment for measuring biopotentials, and possess the benefits of simplicity, low cost, and reliability. However, they have some limitations. The conductive gel dries over time, and is vulnerable to perspiration [6]. This hampers their application in long-term recordings because of their decreasing signal quality over time. Moreover, cleaning and skin preparation, which are necessary in order to yield the best possible conduction, can be time-consuming, particularly when using multiple electrodes. Finally, allergic dermatitis has been reported when using gel electrodes [7]. 

Dry electrodes have been developed to derive biopotentials without any need for skin preparation and conductive gel by using a benign metal. Although some patients developed a metal allergy [8,9], dry electrodes can generally be assumed to improve patient comfort and ensure long-term applicability. However, such electrodes have the disadvantages of higher electrode-to-skin impedance, and are sensitive to movement artifacts [4,6]. Furthermore, metal electrodes in combination with perspiration may degrade or lead to incompatibilities due to galvanic processes that generate unexpected ions [8]. Various designs have been developed to yield similar (electrical) properties to wet electrodes. Novel designs for measuring ECG or electroencephalogram (EEG) signals include needle electrodes [10], dry polymer electrodes [11,12], so-called “epidermal electrodes” [13], and even textile-based dry electrodes [14,15]. The latter are an essential component in the emerging field of adaptable, skin-mounted and wearable electronics [3,16]. The challenge in the development of wearable skin electrodes is to fabricate lightweight, non-interfering, easy-to-handle, biocompatible, flexible electrodes that are capable of delivering good signal quality [5,17]. 

**Figure 1:**
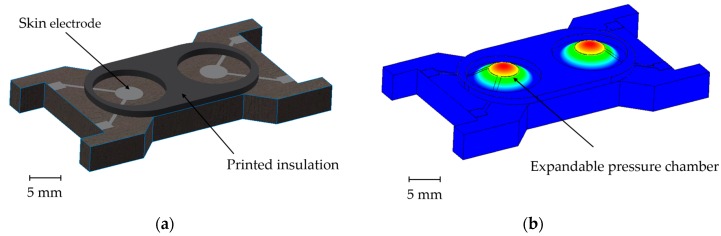
(**a**) Schematic drawing of the electrode module; (**b**) Working principle of the electrode module.

This paper pursues an alternative approach for future healthcare applications: an on-demand electrode-skin contact module. As shown in Figure 1, this comprises a polymeric substrate, electrodes, and an insulation layer, which typically separates the electrodes from skin contact. In case of measurement, the expandable pressure chamber establishes electrode-skin contact by inflating the chambers. After measurement or between measurement intervals, the pressure can be released or the chamber can be evacuated, retracting the electrode from the skin. This approach possesses three advantages: Firstly, the electrode being actively pressed onto the skin ensures good electrical contact. Secondly, with regard to long-term applications, the electrode does not have permanent skin contact, as there are reported issues of rashes during continuous skin contact. The effective electrode-skin contact time is reduced to the time needed for the measurement (seconds), and the electrode is subsequently removed from the skin. If another measurement is needed, contact can be established again. Finally, the module is fully printed, which carries with it all of the advantages of additive manufacturing, including low cost, quickly up scalable and individual fabrication. For instance the shape and number of electrodes is quickly adapted to new requirements. 

This paper demonstrates a first set-up of a pneumatic driven electrode module together with a comparison of screen and inkjet printing on 3D printed substrates to fully additively manufacture electro-pneumatic functions, which can be further applied for designing e.g., microfluidics components such as valves or pumps.

## 2. Additive System Integration

The expendable electrode module was fully additively manufactured using fused deposition modeling (FDM) 3D printing, screen printing, and inkjet printing. 

**Figure 2:**
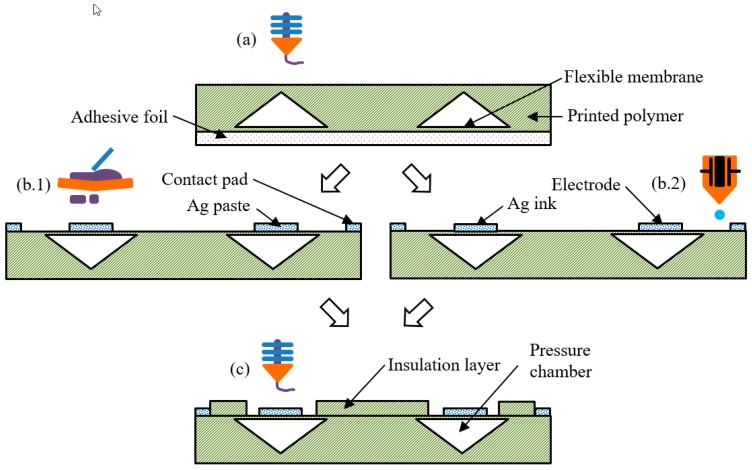
Process steps for printing of the module. (**a**) FDM printing of the basic module; (**b**) Printing of the conductive electrode, (**b.1**) Screen printing of silver paste, (**b.2**) Inkjet-printing of silver ink; (**c**) FDM Printing of the insulation layer.

The manufacturing process of the module is shown in Figure 2. In the first step, (a), the basic module, including pressure chamber and flexible membrane, was printed. The printer Mankati Fullscale XT Plus (Shanghai, China) works with a filament of 2.85 mm in diameter. The flexible filament used was PolyFlex™ from Polymaker LLC (Shanghai, China), and is available in black and white color. The material is thermoplastic polyurethane (TPU) with following properties [18]:

- Shore hardness 95A
- Tensile strength (29 ± 2.8) MPa
- Elongation at break (330.1 ± 14.9) %.

The filament was printed at 230 °C, at a speed of 45 mm/s and a set layer height of 100 µm.

The module is 4 mm high, 40 mm long and 22 mm wide. The setting of the distance between the nozzle and the build plate is the main determinant of the membrane thickness, and was set manually before each print by visual inspection when printing the test structures surrounding the actual model. An arithmetic average surface roughness of about R_a_ = 1.5 µm was achieved by printing on an adhesive foil. The conical shape of the pressure chamber is essential for successful 3D printing without supporting structures. The printer closes the chamber layer by layer, just a little at a time, until it fully closes on top. In the next step, (b), the printing of the electrodes was accomplished by using one of two technologies: screen printing and inkjet printing. For screen printing (Figure 2b.1), polymeric silver paste C5029 from DuPont de Nemours GmbH (Hamm, Germany) and an MPM SPT printer (ITW EAE, Glenview, IL, USA ) was used. The paste was cured for 60 min at 60 °C in a hot air oven according the manufacturer’s process description. The layer height was, on average, 23.1 µm, and the average surface roughness was R_a_ = 4.6 µm. For inkjet printing (Figure 2b.2), NPS-JL silver ink from Harima Chemicals (Tokyo, Kantō, Japan) and a Pixdro LP50 printer (Eindhoven, The Netherlands) with a Dimatix Cartridge system were used. The paste was cured for 60 min at 120 °C in a hot air oven, and has been proven suitable for flexible application in the literature [19]. Two layers were printed, with a resulting average layer height of 1.9 µm and R_a_ = 0.7 µm. The area of the electrodes was 12.6 mm^2^, and two pads were connected to produce a redundant contact. The last step (Figure 2c) was again FDM printing of the insulation layer with a thickness of 0.7 µm using PolyFlex™ on top of the electrodes. Here, the nozzle distance was set not to damage the previously printed electrodes. The connection pads were left uncovered by this layer in order for the electrodes to be able to contact the copper wires, using conductive adhesive H20E from Epoxy Technology Inc. (Billerica, MA, USA). For this first state, the connections were isolated using polymeric tape.

## 3. Methods of Characterization

**Figure 3:**
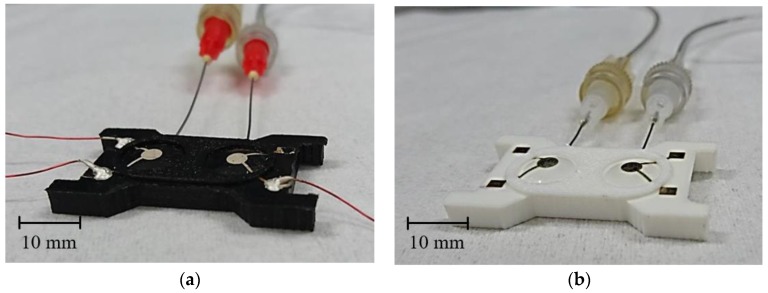
Electrode module with (**a**) screen-printed electrodes and black filament; and (**b**) with inkjet-printed electrodes and white filament.

The electrode module was characterized regarding its suitability for potential long-term skin contact applications. In doing so, both inkjet and screen printing will be considered as possible methods for electrode printing. For practical reasons, and for better illustration, the module with screen-printed electrodes was printed with black filament (Figure 3a), and the inkjet-printed module with white filament (Figure 3b).

### 3.1. Characterization of Dilatation

**Figure 4:**
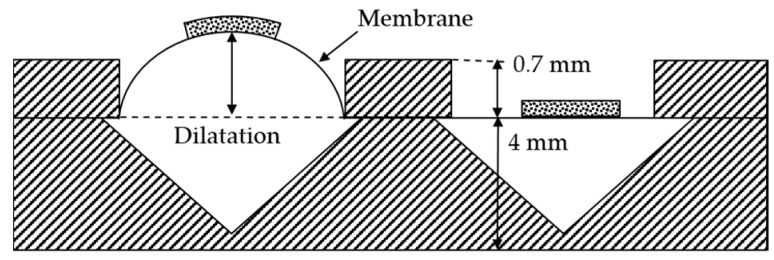
Schematic cross-section of the module in which the left chamber is pressured.

The dilatation of the membrane depends on the pressure applied to the chamber, the thickness of the membrane, and its material properties, as well as the influence of the conductive layer on it. The latter has not been taken into account in this first feasibility study. Due to the manufacturing process, the membrane thickness is subject to variation. To access the pressure chambers of the module from the outside, syringe needles have been used. The connection seals are airtight, and pressure can be applied through syringes of 50 mL volume. The leakage was determined to be 2.4% over a period of time of 60 s at a pressure of 75 kPa. In Figure 4a, the schematic cross-section shows a dilated membrane if the left chamber is pressured. The dilatation was measured using a laser profilometer µscan from NanoFocus AG (Oberhausen, Germany) with the chromatic sensor CLA. The pressure was measured with an analog manometer from Festo AG (Esslingen am Neckar, Germany) with graduation of 100 mbar and a digital DP200 from Mecotec (Gembloux, Belgium). After the experiment, the membrane thicknesses were determined by cutting them out and measuring with µscan. Furthermore, concerning the first reliability forecast, 5 modules with (319 ± 30) µm membrane thickness were tested with 300 pressure and release cycles with a maximum pressure of 100 kPa. At the beginning, and after every 100 cycles, the dilatation was measured at peak pressure using the laser profilometer µscan. The pressure higher than the intended operation pressure was chosen for further skin contact measurements. 

### 3.2. Electrodermal Activity Measurement

For electrical characterization of the electrode module, we exemplarily performed measurements of the electrodermal activity (EDA). EDA is a widely used index for sympathetic nerve activity, which reflects its actions on sweat glands. The more active the nervous system, the higher the skin conductivity, due to its having a higher sweat secretion [20]. 

For this first feasibility study of an alternative approach for an electrode module, we measured 5 healthy subjects. Therefore, both electrode modules were tested at the forearm by pressing them on using approximately 1 N force. The modules were connected to a GSR Amp (ADInstruments, Sydney, Australia), which uses low, constant-voltage AC excitation (22 mV rms @ 75 Hz), thus reducing the electrode polarization artifacts found in DC systems. For reference, the standard MLT116F finger electrodes (stainless steel, electrode area 480 mm^2^, ADInstruments, Sydney, Australia) were attached to the middle and ring finger. For further reference, stainless steel electrodes with an area of 31 mm^2^ were used in the same order at the same measuring spot on the forearm. When measuring, pressure was applied to the pressure chambers of the electrode modules, alternating between 75 kPa (skin contact realized—conduction phase) and −10 kPa (no skin contact—non conduction phase). Modules with membrane thicknesses of (319 ± 30) µm were used, which ensured a proper contact at 75 kPa, while at the same time being far from plastic deformation stress. To assess the reproducibility and stability of the contact, we realized 4 conduction phases (3 times for 10 s and a final phase of 40 s) separated by non-conduction phases of 10 s each. The time was measured using a stopwatch. Steel and finger electrodes were removed manually from the skin with a similar frequency to that at which pressure was released from the screen-printed and inkjet-printed electrodes, in order to evaluate the possible variations in signal course.

## 4. Results

### 4.1. Dilatation

**Figure 5:**
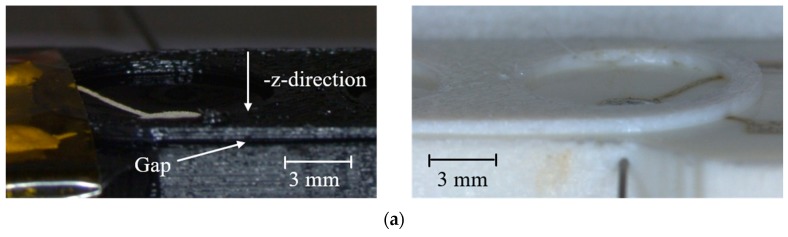

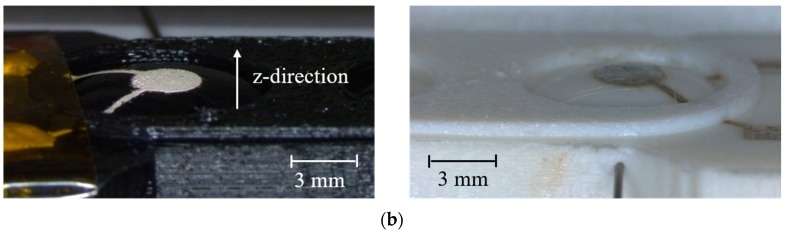
(**a**) View of the screen-printed (**left**) and inkjet-printed (**right**) electrodes with an applied pressure of (**a**) −10 kPa and indrawn membrane (-z-direction); and (**b**) 75 kPa and dilated membrane.

The 3D-printed electrode module was characterized with regard to its suitability for skin electrodes with on-demand contact. Figure 5a shows the screen-printed (left) and the inkjet-printed (right) electrode modules with an applied underpressure of −10 kPa, which leads to indrawn electrodes (-z-direction) and hence the maximum distance between skin and electrodes. In Figure 5b, the modules are depicted with an overpressure of 75 kPa in the pressure chambers. The dilated membrane lifts the electrodes beyond the isolation layer in the z-direction, and is able to establish a contact. The gap shown between substrate and isolation layer indicates alignment issues in the FDM printer.

**Figure 6:**
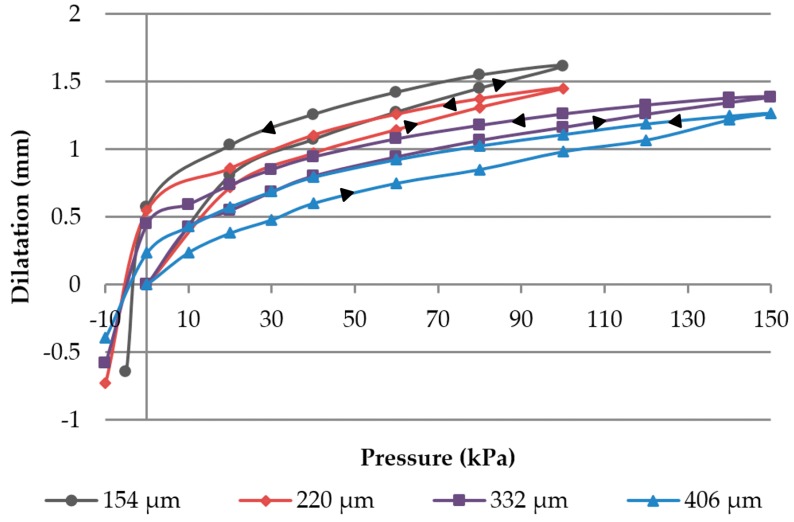
Membrane dilatation of various membrane thicknesses at different pressures (Data from [21]).

Figure 6 shows the dilatation of different membrane thicknesses when the pressure is first increased from 0 kPa to 150 kPa, and afterwards decreased to −10 kPa. During one cycle, only minor viscoelastic behavior of the TPU occurred. This can be noticed in the hysteresis of the dilatation curve, indicated by black arrows, which shows pressure and release direction. The dilatation when increasing pressure (lower curve), compared to the decreasing pressure process (upper curve), is less. The membranes with lower thickness show irreversible deformation at a pressure of above 100 kPa, so the maximum utilized pressure in the measurement of those membranes was lower. Furthermore, a close relation between membrane thickness and dilatation could be observed (the thinner the membrane, the more dilatation at lower pressure). For instance, a 154 µm membrane showed a dilatation of 1.62 mm at 100 kPa. A correlation between membrane thickness and hysteresis was not found after one cycle. As expected, the thickest membrane (406 µm) showed the lowest maximum dilatation. To estimate possible security issues for the electrode module resulting from, e.g., the bursting of the membranes, a pressure of up to 240 kPa was applied. Membranes with 332 µm thickness dilated 1.85 mm at this pressure, but showed major viscoelastic properties when decreasing. Membranes with lower thicknesses were irreversibly plastically deformed, but did not burst.

**Figure 7:**
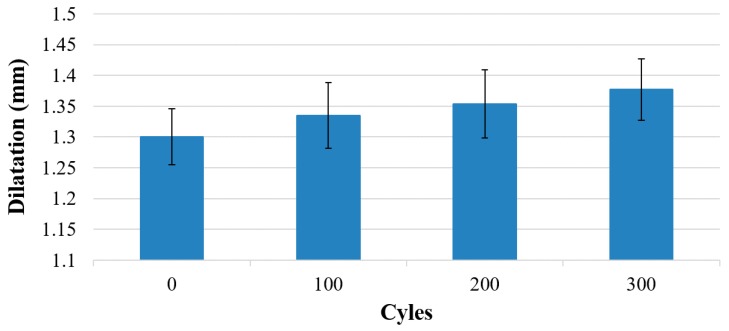
Mean value of the dilatation of 5 membranes of similar printing settings after up to 300 pressure and release cycles (100 kPa) (error bars are 1 standard deviation).

In Figure 7, the dilatation in the z-direction at a pressure of 100 kPa is shown as a function of pressure and release cycles. With increasing cycle numbers, the dilatation also rose. Over 300 cycles, the dilatation rose approximately 5.9%.

### 4.2. Electrodermal Activity Measurement

**Figure 8:**
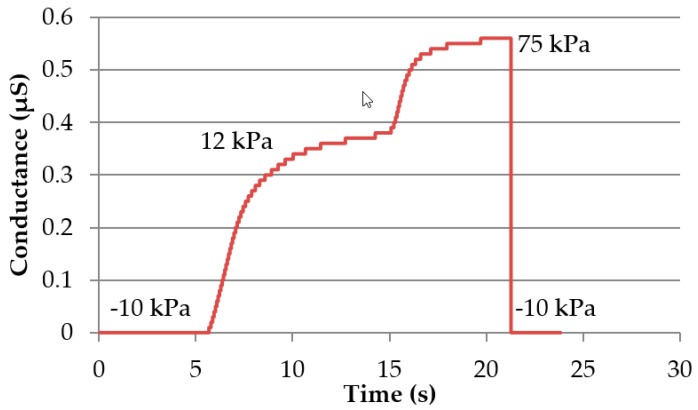
Pressure-dependent conductance on skin of the screen-printed electrode module on one subject (Data from [21]).

The electrical performance of the electrode modules was characterized using EDA measurement (ADInstruments, Sydney, Australia). In Figure 8, the pressure-dependent conductance during on-skin measurements is shown. At a pressure of −10 kPa, no conductance was measured, due to indrawn electrodes. At 12 kPa of pressure, the electrode-skin contact was established, and 75 kPa was the maximum achievable pressure with this set up. Increasing the pressure within the module increases the electrode area, which is pressed onto the skin. In contrast to the soft skin, when pressing the electrodes on rigid surfaces, the pressure dependency was not noticeable. Because of the pressure, the electrode area doesn’t cling to the rigid surface, and hence the contact area doesn’t rise.

**Figure 9:**
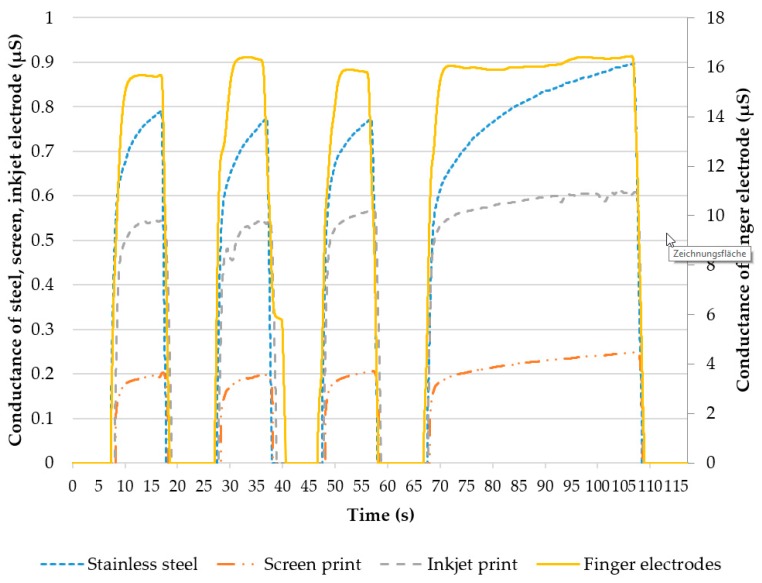
Comparison of screen- and inkjet-printed electrode modules and stainless steel and standard finger electrodes.

Figure 9 shows the EDA measurements for each type of electrode, averaged over all 5 subjects. The finger electrode showed highest conductivity, because fingers and palms contain a higher density of sweat glands compared to the forearm, and the electrode area was the largest. Taking into account that the stainless steel electrode, with the second-largest electrode area, showed the second-highest conductivity, the achieved results are reasonable. The electrode modules both showed lower conductivity in comparison, with the screen-printed electrode being the lowest. Table 1 shows the mean value, standard deviation and variation coefficient for each type of electrode over all four conduction phases. While the absolute amplitude of the finger electrodes was about 10 times higher than the other electrodes, the signal course was qualitatively similar in all 4 types of electrodes. Variation coefficients showed higher variations in the conduction phase for screen-printed, inkjet-printed, and stainless steel electrodes compared to finger electrodes. The longer the conduction, the more the conductance for screen-printed, inkjet-printed and stainless steel electrodes rises (conductance in phase 4 is 11%–23% higher than in phase 1–3), whereas this phenomenon is not observable for the finger electrodes (3%).

## 5. Discussion

This paper presents an alternative approach to yielding electrode-skin contact for future healthcare applications. The presented electrode module was fully printed: substrate, electrodes, and isolation layer. Two different technologies for electrode fabrication, screen- and inkjet-printing, were considered. The module was mechanically and electrically characterized, and showed good performance. The repeatability and reliability, especially of the viscoelastic behavior of the TPU after more than 300 cycles, has to be further evaluated. For this feasibility study, the pressure and release cycles indicated a moderate growing dilatation of 5.9%, which is not expected to have a major impact on possible future applications. This would ensure skin contact. Furthermore, both of the electrode modules, the screen-printed and inkjet-printed, were tested for electrodermal activity measurement and compared to standard electrodes. The difference can be found mainly in the amplitude and the rising peak value during the conductance phase. The reason for the low conductivity can be found in the rising resistance in the conductive tracks, when dilating the membrane. Screen-printed electrodes consist of metal particles in a polymeric matrix that move apart during bending and stretching, leading to increased resistance. Sintered silver ink is likely to produce micro cracks when stretching, but usually shows good performance when bending [21], due to low layer heights, and hence less internal stress. The surface roughness, which increases the effective electrode area, was higher for screen-printed electrodes, and was therefore unlikely to contribute a dominant effect to the EDA results obtained. The characterization of resistance behavior when inflating the module will be the content of future works.

Rising peak values in all electrodes apart from the finger electrodes might be attributed to the softer tissue at the forearm. Repeated application of pressure and the electrode’s contact might lead to some tissue adaptation to the electrode’s shape. Due to the greater size and different tissue properties, this effect is likely to vanish at the finger. However, our physiological measurements had a limited extent and were intended to prove feasibility only. Future works will include more elaborate testing, including physiological stimuli, in order to characterize the sensitivity of our electrode modules. Further, future work will characterize the reproducibility of the manufacturing process in detail. All in all, the on-demand electrode module represents a low-cost design, with all the advantages of 3D printing, and therefore might be suitable for consideration for disposable electrodes or consumer electronics.

## Figures and Tables

**Table 1:** Mean value, standard deviation and variation coefficient of the EDA measurement.

Electrode	Conductance Average in µS	Standard Deviation in µS	Variation Coefficient
Screen printed	0.205	0.030	0.146
Inkjet printed	0.552	0.064	0.116
Stainless steel	0.754	0.097	0.128
Finger electrode	15.6	1.27	0.082
